# Targeted next generation sequencing with an extended gene panel does not impact variant detection in mitochondrial diseases

**DOI:** 10.1186/s12881-018-0568-y

**Published:** 2018-04-07

**Authors:** Morgane Plutino, Annabelle Chaussenot, Cécile Rouzier, Samira Ait-El-Mkadem, Konstantina Fragaki, Véronique Paquis-Flucklinger, Sylvie Bannwarth

**Affiliations:** 1 Université Côte d’Azur, CHU de Nice, Inserm, CNRS, IRCAN, Nice, France; 2 IRCAN UMR CNRS 7284/INSERM U1081, Medicine School, 28 av de Valombrose, 06107 Nice cedex 2, France

**Keywords:** Mitochondrial disorders, Next generation sequencing, Targeted panel, Exome

## Abstract

**Background:**

Since the advent of next generation sequencing (NGS), several studies have tried to evaluate the relevance of targeted gene panel sequencing and whole exome sequencing for molecular diagnosis of mitochondrial diseases. The comparison between these different strategies is extremely difficult. A recent study analysed a cohort of patients affected by a mitochondrial disease using a NGS approach based on a targeted gene panel including 132 genes. This strategy led to identify the causative mutations in 15.2% of cases. The number of novel genes responsible for respiratory chain deficiency increases very rapidly.

**Methods:**

In order to determine the impact of larger panels used as a first screening strategy on molecular diagnosis success, we analysed a cohort of 80 patients affected by a mitochondrial disease with a first mitochondrial DNA (mtDNA) NGS screening and secondarily a targeted mitochondrial panel of 281 nuclear genes.

**Results:**

Pathogenic mtDNA abnormalities were identified in 4.1% (1/24) of children and 25% (14/56) of adult patients. The remaining 65 patients were analysed with our targeted mitochondrial panel and this approach enabled us to achieve an identification rate of 21.7% (5/23) in children versus 7.1% (3/42) in adults.

**Conclusions:**

Our results confirm that larger gene panels do not improve diagnostic yield of mitochondrial diseases due to (i) their very high genetic heterogeneity, (ii) the ongoing discovery of novel genes and (iii) mutations in genes apparently not related to mitochondrial function that lead to secondary respiratory chain deficiency.

**Electronic supplementary material:**

The online version of this article (10.1186/s12881-018-0568-y) contains supplementary material, which is available to authorized users.

## Background

Mitochondrial disorders (MD) take part of a group of rare diseases, characterized by an impairment of the mitochondrial respiratory chain (RC), with a prevalence of 1:5000 live births [1]. Deficiency of the mitochondrial RC is responsible for the lack of ATP production, which provides energy in each cell through oxidative phosphorylation (OXPHOS). The diagnosis of such diseases is challenging because of extreme phenotypic heterogeneity, variable age of onset and different modes of inheritance. Among these diseases, some affect a single specific organ (like Leber Hereditary Optic Neuropathy, LHON), but a majority of them involve multiple organ systems. The clinical spectrum is very wide, from mild clinical features such as Chronic Progressive External Ophtalmoplegia (CPEO) to very severe neurologic impairment such as Leigh Syndrome (LS). The common clinical features of mitochondrial diseases include ptosis, ophtalmoplegia, myopathy, cardiomyopathy, sensorineural deafness, optic atrophy, pigmentary retinopathy and diabetes mellitus. Encephalopathy, epilepsy, cerebellar ataxia, axonal neuropathy, migraine, stroke-like episodes, cognitive impairment and movement disorders are mainly found in patients presenting with neurological symptoms.

Most of the proteins required for structure, biogenesis and function of mitochondria are encoded by nuclear genes (nDNA) but 13 essential subunits of RC complexes are encoded by the mitochondrial genome. Human mitochondrial DNA (mtDNA) is a circular double-stranded molecule, constituted by 16,569 base pairs in size, encoding 13 respiratory chain subunits, 22 tRNAs and 2 rRNAs. MtDNA is present in multiple copies within the mitochondria of each cell [2]. Although mitochondria possess their own genome, they need nuclear genes to encode proteins for their biogenesis including mtDNA maintenance, mitochondrial dynamics (fusion and fission), coenzyme Q10 biosynthesis, assembling of the respiratory chain complexes, activity and turnover. More than 250 nuclear genes have already been linked to play a role in mitochondrial disorders and the list of candidate genes is growing up as over 1500 genes have been identified controlling mitochondrial structure and function [3, 4]. Finding the pathogenic variants by molecular genetic testing confirms the diagnostic, and provides to the geneticist the ability to deliver genetic counselling. However, responsible genes remain to be identified in most patients suspected with a mitochondrial disease. Recently, Next Generation Sequencing (NGS) has improved the efficiency of mutation discovery and facilitated the molecular routine diagnosis of such diseases in term of money and time spent [5, 6].

In a recent study, Legati and colleagues analysed a cohort of patients, affected by a mitochondrial disease mostly characterized by early onset, using a combined NGS approach based on a targeted gene panel and whole exome sequencing (WES) [6]. Their custom-made targeted mitochondrial panel included 132 genes. It allowed to identify the causative mutations in 15.2% of cases. The authors then identified the causative molecular abnormalities in 6 out of 10 patients tested by WES. In order to determine the impact of larger panels on molecular diagnosis success, we analysed a cohort of 80 patients affected by a mitochondrial disorder. A mtDNA disease was identified in 15 patients (18.7%) including 1 child out of 24 (4.1%) and 14 adults out of 56 (25%). In a second step, the remaining 65 patients were analysed with a targeted mitochondrial panel of 281 genes allowing to obtain a detection rate of 12.3% with 21.7% (5/23) in children and of 7.1% (3/42) in adult patients.

## Methods

### Clinical and biochemical investigation of patients

Eigthy patients (38 males and 42 females; 24 children (9 with age onset < 1; median age of the cohort =1 ± 3.3 years) and 56 adults (median age of the cohort = 58 ± 14.3 years) diagnosed as affected by mitochondrial disease through the analysis of clinical, biochemical and histological data, were included in this cohort (Table 1). All have been referred to the National Centre of Mitochondrial Diseases (CHU Nice, France), also certified by the European Network of reference centers for rare neuromuscular diseases (EURO-NMD). Histological analysis was available in 63 cases out of 80 with muscle biopsies evocative of a mitochondrial myopathy in 45 patients out of 63 (10/19 children and 35/44 adults). Based on the biochemical data obtained in either muscle, liver, fibroblasts or both and available in the large majority of patients (59 out of 80), we identified isolated defect in complex I (*n* = 4; 2/21 children and 2/38 adults), complex II (*n* = 1; 1/21 child), complex III (*n* = 6; 6/38 adults), complex IV (*n* = 3; 2/21 children and 1/38 adult) and multiple defects (*n* = 10; 6/21 children and 4/38 adults) (Table 1). Informed consent for diagnostic and research studies was obtained for all subjects in accordance with the Declaration of Helsinki protocols.

**Table 1:** List of the patient cohort

Patient N°	Age-range at investigations (years)	Sex	Muscle histology	Biochemical defect	mtDNA deletions	Clinical presentation
1	< 1	M	N	↓CIII, IV	No	Encephalopathy with spastic dystonia
2	< 1	F	N	N	No	Leigh syndrome, growth retardation, dystonia
3	< 1	F	N	↓CI	No	Encephalopathy, hepatomegaly, epilepsy, leukodystrophy
4	< 1	M	lipidosis	N(m) + impaired assembly CI, N(l)	No	Leigh syndrome
5	< 1	F	N	↓CI	No	Spastic tetraparesia, growth retardation
6	< 1	F	NA	NA	NA	Congenital cataract, microphtalmia, hypotonia, myocardic dysfunction
7	< 1	M	N	Multi	No	Leigh syndrome, growth retardation, myoclonic epilepsy, chronic diarrhea, vascular purpura, lactic acidosis, death at 3 years-old
8	< 1	M	RRF lipidosis	N	No	Neonatal hypotonia, growth retardation, myopathy, cardiopulmonary failure, methylglutaconic aciduria
9	< 1	M	NA	↓CII, III, IV	NA	Hypertrophic cardiomyopathy, epileptic encephalopathy, methylglutaconic aciduria
10	1–16	F	lipidosis	N	No	Dilated cardiomyopathy, cerebellar ataxia
11	1–16	M	↓ COX activity	↓CIV	No	Hypotonia, hypertrophic cardiomyopathy, hyperlactatemia
12	1–16	M	NA	N (f)	No	Psychomotor delay, cerebellar ataxia,
13	1–16	M	NA	↓CII and impaired assembly CII (f)	No	Psychomotor regression, spastic quadriparesis, leukodystrophy
14	1–16	F	COX-	N	No	Leigh syndrome without regression, encephalopathy, dystonia, hyperlactatemia
15	1–16	M	COX-	NA	NA	Myopathy
16	1–16	M	N	↓CII, IV	No	Leigh syndrome with regression, epilepsy, ptosis
17	1–16	M	↓COX activity lipidosis	N	No	Migraine, Stroke-like episodes, psychomotor delay
18	1–16	F	N	↓CI, II, III, IV	No	Axonal neuropathy, cerebellar ataxia with cerebellar atrophy, deafness
19	1–16	M	N	↓CI, IV	No	Psychomotor delay, moderate and late-onset Leigh syndrome, spastic paraparesia
20	1–16	F	lipidosis	N (m, f)	Yes	Episodic metabolic encephalopathy, deafness
21	1–16	M	COX-	N	Yes	Dilated cardiomyopathy, growth retardation, diabetes mellitus
22	1–16	M	COX- SDH- lipidosis	↓CIV	No	Motor delay, refractory status epilepticus, regression, pyramidal and extrapyramidal syndrome
23	1–16	M	N	N, impaired assembly CIII (m), CV(l), N(f)	No	Stroke-like episodes, dystonia, myalgia, intellectual disability
24	1–16	F	NA	NA	NA	Fahr syndrome + clinical pseudostroke
25	> 16	M	N	N (m, f, l)	No	Myoclonic epilepsy, ptosis
26	> 16	M	N	↓CIII	No	Cerebellar ataxia, stroke-like episodes
27	> 16	F	N	↓CII, III	No	Psychiatric disorder, distal weakness, vertical supranuclear gaze palsy, dystonia
28	> 16	M	COX- lipidosis	N	No	Rhabdomyolysis
29	> 16	F	N	NA	Yes	CPEO, sensorineural deafness, migraine
30	> 16	F	NA	NA	NA	Optic atrophy
31	> 16	M	NA	NA	NA	Optic atrophy
32	> 16	F	RRF COX-	N	No	Sensorineural hypoacusia, CPEO, cachexia
33	> 16	F	NA	N, impaired assembly CV	No	Leigh syndrome
34	> 16	F	lipidosis	↓CIII	No	Spinal muscular atrophy syndrome
35	> 16	F	COX-	N	Yes	Sensory peripheral neuropathy, dysautonomia
36	> 16	M	RRF COX-	N (f)	Yes	Ptosis, exercice intolerance, lipomatosis, dysphonia
37	> 16	F	N	↓CIII	Yes	Myalgia, exercise intolerance, diabetes, hypoacusia
38	> 16	M	COX -	NA	Yes	Peripheral neuropathy, diabetes
39	> 16	M	COX-	↓CIII	NA	Myalgia, axial myopathy, ptosis
40	> 16	M	NA	NA	NA	Optic atrophy
41	> 16	F	N	↓CII, III	Yes	Sensory ataxic neuropathy, optic neuropathy
42	> 16	F	RRF COX-	NA	Single deletion	Kearn-Sayre Syndrome
43	> 16	F	RRF COX- lipidosis	N	Yes	CPEO, motor-sensory demyelinating neuropathy
44	> 16	F	NA	NA	NA	Diabetes, sensorineural deafness, pattern macular dystrophy, myalgia, nephropathy
45	> 16	M	RRF Mitochondrial aggregates	NA	Yes	Cerebellar ataxia, dilated cardiomyopathy, peripheral neuropathy, renal insufficiency, deafness
46	> 16	M	NA	NA	NA	Optic atrophy
47	> 16	F	RRF lipidosis	N	Yes	Proximal myopathy, stroke-like episodes, psychiatric disorder, cognitive impairment
48	> 16	F	lipidosis	N	Yes	Myalgia, exercise intolerance, rhabdomyolysis
49	> 16	M	N	↓CIII, IV, V	No	Axonal neuropathy, ophtalmoplegia, tremor, lipomatosis, deafness, cognitive impairment
50	> 16	F	N	↓CIII	Yes	CPEO
51	> 16	F	NA	NA	NA	Bilateral optic atrophy
52	> 16	M	N	↓CII, III, IV	Yes	Exercise intolerance
53	> 16	M	RRF COX-	N	Yes	Exercise intolerance, epilepsy, ptosis, peripheral neuropathy, extra-pyramidal syndrome
54	> 16	F	RRF	NA	Single deletion	Ptosis, proximal myopathy
55	> 16	M	RRF COX- lipidosis	NA	NA	Hypertrophic cardiomyopathy, hypoacusia, strokes
56	> 16	F	RRF COX-	N	Yes	CPEO, deafness, proximal myopathy
57	> 16	F	lipidosis	N	Yes	Axonal and sensory ataxic neuropathy, deafness, retinitis pigmentosa
58	> 16	M	NA	NA		Diabetes, deafness
59	> 16	M	RRF COX- lipidosis	N	No	Myopathy, ptosis, dilated cardiomyopathy, dysphagia
60	> 16	F	COX- lipidosis	N	No	Ptosis, myalgia, exercise intolerance
61	> 16	M	COX-	N	Yes	Exercise intolerance, myalgia, rhabdomyolysis
62	> 16	F	NA	NA		Diabetes, deafness, cerebellar ataxia, hypertrophic cardiomyopathy
63	> 16	M	NA	NA	No	Hypertrophic cardiomyopathy, deafness
64	> 16	M	Mitochondrial aggregates	N	Yes	Cerebellar ataxia, CPEO
65	> 16	F	COX-	NA	Yes	Dementia, axial myopathy, stroke-like episodes
66	> 16	M	COX-	↓CIV + impaired assembly CIV	No	CPEO, dysphagia
67	> 16	M	COX- lipidosis	↓CI	Yes	Cerebellar ataxia, myoclonic epilepsy, cataract, deafness, hyperlactatemia
68	> 16	M	COX-	N	Yes	CPEO, dysphagia
69	> 16	F	COX-	↓CIII	Yes	Cerebellar syndrome, hepatic steatosis
70	> 16	F	NA	NA	NA	CPEO, stroke-like episodes
71	> 16	F	COX-	N	Yes	Axonal and sensory ataxic neuropathy, cachexia, deafness
72	> 16	F	RRF COX-	↓CI and quinones	Yes	Unilateral ptosis with familial history of autosomal dominant CPEO
73	> 16	F	COX-	N	Yes	Myopathy
74	> 16	F	NA	NA	NA	Sensory ataxic neuropathy, optic neuropathy, cognitive impairment, white matter hyperintensities
75	> 16	F	RRF COX-	N	Yes	Axonal and sensory ataxic neuropathy, deafness, cardiac conduction block
76	> 16	M	COX-	N	Yes	CPEO, ataxia
77	> 16	M	COX-	hyperactivity CII, III, IV and CI + III, II + III	Yes	Peripheral neuropathy, fronto temporal dementia, Paget disease
78	> 16	M	RRF COX-	N, impaired assembly CI	Yes	Axonal and sensory ataxic neuropathy, rhabdomyolysis
79	> 16	M	COX-	N	Yes	CPEO, dysphagia, deafness
80	> 16	M	RRF COX-	N	Yes	Ptosis, dysphagia, cerebellar ataxia, hypoacusia, exercise intolerance

Biochemical analyses were performed using muscle biopsies (m) or other tissues (f: fibroblasts, l: liver)

↓: decreased; CI, CII, CIII, CIV, CV: respiratory chain complexes; Multi: decreased of all respiratory chain complexes; *N* normal, *COX-* COX negative fibers, *RRF* ragged-red fibers, *SDH* succinate deshydrogenase, *CPEO* chronic progressive external ophtalmoplegia, *NA* not available

### Molecular genetics

#### mtDNA analysis

The identification of mtDNA single deletions and point mutations has been performed by using XL-PCR and NGS protocols, respectively [7]. The presence of mtDNA deletions was confirmed by Southern bot analysis [8].

#### Custom targeted panel analysis

We designed a custom panel of genomic regions corresponding to 281 genes, selected in 2016 to be already involved in mitochondrial disorders (NIH Genetic Testing Registry) or to be candidate genes (Additional file 1: Table S1). We designed RNA probes to capture the transcribed sequences of genes (exons and exon/intron junctions) with Agilent SureSelect kit. 1 μg of genomic DNA was fragmented and adaptors were added in a single enzymatic step by the library builder (Thermofisher Scientific). The adaptor-tagged DNA library was purified and amplified. 750 ng of each library was hybridized using SureSelect capture library overnight at 65 °C. The resulting libraries were recovered using streptavidin beads and a post-capture PCR amplification was carried out. Libraries were pooled, emulsion PCR, enrichment and loading of template-positive ion sphere particles were performed on an IonChef system. Ion PI chips V3 were sequenced on the Ion Proton, using Ion PI Hi-Q sequencing kit. The sequences were aligned against the human reference sequence (GRCh37/hg19) using Torrent Suite Software 5.0.4. Variant calling was then performed using variant caller version 5.0.4.0. Annotation and filtering of the variants were accomplished by submitted them to Ion Reporter Software version 5.2. Filtering was carried out by applying a series of steps: variants with a minor allele frequency (MAF) < 1% in the 1000 genomes project or in the 5000 exomes european-american (NHLBI ESP) were kept. We focused on predicted missense, frame-shift, stop-gain or stop-loss, and splice-site variants. For remained variants in the final list, we also checked the prediction score in Polyphen 2 (http://genetics.bwh.harvard.edu/pph2/), SIFT (http://sift.jcvi.org/) and their interpretation in MutationTaster (http://www.mutationtaster.org/).

### Validation of variants identified by NGS

Variants identified by NGS were validated by Sanger sequencing. Coding regions with exon/intron junctions were amplified through PCR (primers available upon request). PCR products were sequenced using an ABI Prism 3100XL apparatus (Applied Biosystems). The chromatograph traces were analyzed using Sequencing Analysis software.

## Results

### Analysis of the mitochondrial genome

During the first 3 months of 2016, 80 patients have been referred to the National Centre of Mitochondrial Diseases in Nice. A mtDNA mutation was identified in 15 patients (1/24 children and 14/56 adults) by NGS analysis (Table 2). The m.1555A > G mutation was found in a 15 year-old patient (N°24) presenting with a pseudo-stroke episode associated with Fahr syndrome. The same mutation was carried by one adult patient (N°58) and was responsible for diabetes and deafness. The m.3243A > G mutation was identified in 5 adult individuals (N°28, 44, 55, 62, 63) and was responsible for symptoms including diabetes, hypertrophic cardiomyopathy, deafness or rhabdomyolysis. Three patients (N°31, 40, 46), presenting with Leber’s hereditary optic neuropathy (LHON), carried the m.11778G > A mutation. A 31 year-old patient (N°30) also had an optic neuropathy associated with the m.3460G > A pathogenic variant. The m.8993 T > C mutation was responsible for an infancy-onset Leigh syndrome in a 34 years-old patient (N°33) and the m.5703G > A mutation was identified in an adult patient (N°32) presenting with hypoacusia, CPEO and cachexia. Two additional patients (N°42 and 54), presenting with Kearns Sayre syndrome or mitochondrial myopathy, carried a heteroplasmic mtDNA deletion in muscle (Table 2). Thirty adult patients and 2 children carried mtDNA multiple deletions in muscle.

**Table 2:** Pathogenic variants identified by the NGS analysis in this study. Variants identified in mtDNA

Patient N°	Age-range onset (years) / sex	Pathogenic variant	Tissue	Clinical presentation
24	1–16 / F	m.1555A > G homoplasmic	blood, urine	Fahr syndrome + clinical pseudostroke
28	> 16 / M	m.3243A > G heteroplasmic	muscle	Rhabdomyolysis
30	> 16 / F	m.3460G > A homoplasmic	blood	Optic atrophy
31	> 16 / M	m.11178G > A homoplasmic	blood	Optic atrophy
32	> 16 / F	m.5703G > A heteroplasmic	urine, buccal	Sensorineural hypoacusia, CPEO, cachexia
33	Infancy / F	m.8993 T > C homoplasmic	muscle	Leigh syndrome
40	> 16 / M	m.11178G > A homoplasmic	blood	Optic atrophy
42	> 16 / F	Single deletion	muscle	Kearn-Sayre Syndrome
44	> 16 / F	m.3243A > G heteroplasmic	buccal, blood	Diabetes, sensorineural deafness, pattern macular dystrophy, myalgia, nephropathy
46	> 16 / M	m.11178G > A homoplasmic	blood	Optic atrophy
54	> 16 / F	Single deletion	muscle	Ptosis, proximal myopathy
55	> 16 / M	m.3243A > G heteroplasmic	blood, muscle	Hypertrophic cardiomyopathy, hypoacusia, strokes
58	> 16 / M	m.1555A > G heteroplasmic	blood	Diabetes, deafness
62	> 16 / F	m.3243A > G heteroplasmic	blood, urine	Diabetes, deafness, cerebellar ataxia, hypertrophic cardiomyopathy
63	> 16 / M	m.3243A > G heteroplasmic	blood	Hypertrophic cardiomyopathy, deafness

### Analysis of the nuclear genome

We used a targeted gene panel to analyze the coding sequences as well as the exon/intron junctions of 281 genes in the remaining 65 patients who were negative for mtDNA screening. The regions were captured with 34,839 RNA probes corresponding to a total amount of the targeted genomic regions of 490 Kb. An average number of 9,333,126 reads was produced per sample. On average, 100% of targeted bases were covered and 99.3% of targeted bases exceeded 20X coverage threshold. The Torrent Variant Caller plug-in annotated an average number of 312 variants per sample (SNVs and INDELs).

Depending on the clinical and familial history, we focused on autosomal recessive, dominant or X-linked mode of disease inheritance. If an autosomal recessive mode of inheritance was suspected, we analyzed specifically homozygous and compound heterozygous variants. In cases of only one heterozygous variant found, we performed extended systematic NGS coverage analysis in search of a second allelic variant, including genomic deletions. When the mode of inheritance was unknown or compatible with autosomal dominant or X-linked transmission, single heterozygous variants were also considered.

### Pathogenic variants

After variant calling and filtering analysis, we identified pathogenic variants responsible for the disease in 8 patients out of 65 (12.3%), including 5/23 children (21.7%) and 3/42 (7.1%) adults (Table 3).

**Table 3:** Pathogenic variants identified by the NGS analysis in this study. Variants identified by the NGS panel

Patient N°	Age-ranges onset (years) / sex	Gene	Nucleotide change	Protein change	Trait	Concordant phenotype	ExAC frequency	SIFT score	Polyphen 2	Variant reported	Notes	References
6	NN / F	*AGK*	c.412C > T / c.412C > T	p.Arg138*	AR	Yes	0	NA	NA	Yes	Csg; parents: htz	Mayr et al., 2012 [36]
7	NN / M	*ETHE1*	c.608C > T / c.554 T > G	p.Ser203Phe / p.Leu185Arg	AR	Yes	< 0.01% < 0.01%	0.0 0.0	0.975 1	No Yes	Parents: htz	Tiranti et al., 2005 [37]
10	NN / F	*DNAJC19*	c.51delT / c.51delT	p.Phe17Leufs*10	AR	Yes	0	NA	NA	No	–	–
13	1–16 / M	*SDHAF1*	c.164G > C / c.164G > C	p.Arg55Pro	AR	Yes	0	0.0	1.0	Yes	Parents: htz	Ghezzi et al., 2009 [38]
15	1–16 / M	*TK2*	c.343C > T / c.323C > T	p.Leu115Phe / p.Thr108Met	AR	Yes	0 < 0.01%	0.0 0.2	1 1	No Yes	Parents: htz	Béhin et al., 2012 [39]
29	> 16 / F	*TWNK*	c.1363A > G	p.Met455Val	AD	Yes	0	0.0	0.996	No	–	–
43	> 16 / F	*TYMP*	c.1112 T > C / c.1112 T > C	p.Leu371Pro	AR	Yes	0	0.18	0.999	Yes	–	Kocaefe et al., 2003 [40]
68	> 16 / M	*OPA1*	c.1892_1893delAT	p.His631Argfs*3	AD	Yes	0	NA	NA	Yes	–	Ferré et al., 2009 [41]

Mutation Taster predicted all variants to be disease causing; *NN* neonatal, *AR* autosomal recessive, *AD* autosomal dominant, *htz* heterozygous, *NA* not available, *Csg* consanguinity

*stop codon

Four patients (N°6, 10, 13, 43) carried homozygous pathogenic variants within the *AGK, DNAJC19, SDHAF1 and TYMP* genes whereas 2 (N°7, 15) carried compound heterozygous pathogenic variants within *ETHE1* and *TK2* respectively. Two patients (N°29, 68) carried one heterozygous causative mutation, within *TWNK or OPA1*, responsible for a dominant disease. Among the 10 different identified pathogenic variants, six corresponded to already known causative mutations. Four were predicted pathogenic variants in genes responsible for mitochondrial diseases. The phenotypes of these eight first patients overlapped with the clinical presentations previously described for the corresponding genes and segregation studies, available in 4 cases, were concordant with the pathogenicity of the identified variants (Table 3).

### Variants of uncertain significance (VUS)

We also identified VUS in 7 patients. One male adult patient (N°66) carried a novel hemizygous variant (c.893G > A; p.Arg298Gln) in the *AIFM1* gene, localized on Xq26.1, and encoding the apoptosis-inducing factor (AIF) (Table 4). Since the age of 60, the patient had CPEO associated with dysphagia but without any other associated symptom (Table 1). His mother presented exactly the same clinical signs with same onset but she was not available for testing. At the age of 69, muscle biopsy revealed COX-negative fibers with a complex IV deficiency by spectrophotometry. Blue native PAGE analysis revealed an assembly defect or increased instability of complex IV. The c.893G > A variant is not frequent in ExAC database (< 0.01%) (5/87658 tested alleles including 2 in a hemizygous state in male individuals). It affects a highly-conserved residue in the NADH-binding domain and is predicted as probably pathogenic. Mutations in *AIFM1* reported so far cause a progressive disorder affecting the muscles and the nervous system with a more severe phenotype than the one presented by our patient [9]. Without further functional analyses, it will be difficult to definitively confirm the deleterious consequences of the identified variant.

**Table 4:** Variants identified by the NGS panel in this study. Variants of Uncertain Significance

Patient N°	Age-ranges onset (years) / sex	Gene	Nucleotide change	Protein change	Trait	Concordant phenotype	ExAC frequency	SIFT score	Polyphen 2	Variant reported	Notes	References
34	1–16 / F	*KIF1B*	c.4682G > A	p.Cys1561Tyr	AD	Yes	< 0.01%	0.02	0.987	No	–	–
35	> 16 / F	*POLG2*	c.390-2A > C	–	AD	Yes	1	–	–	No	+ c.1105A > G htz (polymorphism)	–
64	> 16 / M	*SYNE1*	c.23315G > A / c.15337G > A	p.Arg7772Gln / p.Val5113Ile	AR	Yes	< 0.01% < 0.01%	0.01 0.29	0.956 0.968	No Yes	c.15337G > A htz (patient’s daughter)	Neubauer et al., 2017 [17]
66	1–16 / M	*AIFM1*	c.893G > A	p.Arg298Gln	X-linked	+/−	< 0.01%	0.63	0.402	No	Mild phenotype	Ardissone et al., 2015 [9]
67	> 16 / M	*DNA2*	c.2862G > C	p.Leu954Phe	AD	Yes	0	0.0	0.669	No	Severe phenotype	–
71	> 16 / F	*KIF5A*	c.1248A > T	p.Lys416Asn	AD	Yes	0	0.01	0.935	No	–	–
77	> 16 / M	*KIF5A*	c.2354A > G	p.Glu785Gly	AD	Yes	0	0.0	0.999	No	–	–

We identified 2 subjects carrying a heterozygous variant in *KIF5A,* which encodes a kinesin-like protein. Both patients presented with a sensory axonal polyneuropathy (Table 1). The first case, a 70 years-old patient (N°71), also had ataxia, hearing loss and cachexia. COX-negative fibers were found in muscle but without respiratory chain deficiency. The second case (N°77) was a 77 years-old patient who suffered from frontotemporal dementia and Paget disease. The 2 variants, c.1248A > T; p.Lys416Asn, and c.2354A > G; p.Glu785Gly, respectively, are not found in ExAC databases and in silico analysis predicts them to be probably pathogenic (Table 4). The corresponding amino acids are located in the coiled-coil domain and are conserved over species. Mutations in *KIF5A* are described in a wide clinical spectrum from hereditary spastic paraplegia (HSP) 10 to axonal neuropathy [10] and was recently implied in early-onset phenotype with severe myoclonus and evidence of mitochondrial dysfunction [11].

We also identified a novel heterozygous variant, c.4682G > A; p.Cys1561Tyr in another kinesin family member (*KIF1B*) (Table 4). The patient (N°34) presented with an early-onset disease including frequent falls and lower limb weakness. At 16 years of age, he developed a tetraparesia during an infectious episode with progressive incomplete recovery. At 43 years-old, he has lower limb weakness with areflexia, and jaw muscle weakness. Electromyogram study showed evidence of anterior horn cell involvement and we found a complex III deficiency in muscle. The missense variant affects a highly conserved amino acid located in the PH (Pleckstrin Homology) domain, is not frequent in ExAC database (< 0.01%) and is predicted to be deleterious (Table 4).

Heterozygous pathogenic variants in *DNA2* have been identified in adult-onset mitochondrial myopathy with mtDNA instability [12]. We found a novel *DNA2* heterozygous variant, c.2862G > C; p.Leu954Phe in a patient presenting with cerebellar ataxia, myoclonic epilepsy, cataract and bilateral hearing loss (Patient N°67) (Table 4). Muscle biopsy revealed histological signs of mitochondrial myopathy with low level in complex I and mtDNA multiple deletions. The variant, localized in the helicase domain, is not found in ExAC database and in silico analysis predicts it as pathogenic (Table 4).

We identified 2 heterozygous variants in *POLG2* in a 38 year-old patient (N°35) presenting with sensory neuropathy and multiple mtDNA deletions (Table 4). The c.1105A > G; p.Arg369Gly variant was firstly being classified as functionally pathogenic [13]. It was then reclassified by ClinVar as variant of undetermined signification. More recently, this variant has been identified in homozygous state in control individuals and is now considered as likely benign [14]. The second variant (c.390-2A > C) is supposed to have an effect on splicing by in silico analysis (http://www.mutationtaster.org/). Mutations that affect splicing and POLG2 expression have previously been described in patients [15, 16]. Thus the c.390-2A > C splice acceptor variant in *POLG2* we identified is consistent with previous *POLG2* pathogenic variants responsible for mtDNA deletions and late-onset mitochondrial disease.

Two compound heterozygous variants, c.23315G > A; p.Arg7772Gln and c.15337G > A; p.Val5113Ile, were found within *SYNE1* (spectrin repeat-containing nuclear envelope protein 1) in a 46-year old patient (N°64) presenting with a cerebellar syndrome and CPEO. DNA samples from his parents were not available but his asymptomatic daughter carried one variant only c.15337G > A; p.Val5113Ile, that had been previously described (Table 4) [17]. There was mitochondrial aggregation with mtDNA deletions but no RC deficiency in the patient’s muscle. The 2 variants are rare (< 0.01% in EXAC) and predicted to be deleterious. Disease-causing *SYNE1* variants were previously reported in a large clinical spectrum with bi-allelic mutations responsible for SCAR8 phenotype including pure cerebellar atrophy, ataxia and dysarthria, with variable age at onset of symptoms (6–50 years) [18].

### Misannotated mutations and likely benign variants

In several samples, we found variants that have previously been annotated as pathogenic, but the current patients did not present with symptoms attributed to those mutations. For instance, a known pathogenic variant c.1987C > T; p.Arg663Cys in *MFN2*, which encodes a mitochondrial GTPase mitofusin protein, was found in a 14-year old patient (N°23) (Table 5) [19]. Another heterozygous variant c.1085C > T; p.Thr362Met in the same gene was identified in another patient at 58 years of age (N°52). Mutations in *MFN2* are responsible for Charcot-Marie-Tooth type 2A (CMT2) and hereditary motor and sensory neuropathy VI [20, 21]. Both patients, however, did not present with peripheral neuropathy.

**Table 5:** Variants identified by the NGS panel in this study. Misannotated or probably non-pathogenic variants

Patient N°	Age onset (years) / sex	Gene	Nucleotide change	Protein change	Trait	Concordant phenotype	ExAC frequency	SIFT score	Polyphen 2	Variant reported	Notes	References
2	NN / F	*PC*	c.715A > G /?	p.Ile239Val	AR	No	< 0.01%	0.28	0.04	No		–
22	1–16 / M	*OPA3*	c.229G > A /?	p.Ala77Thr	AD / AR	No	0	0.12	1.0	No	Mother: htz	–
23	1–16 / M	*MFN2*	c.1987C > T	p.Arg663Cys	AD	No	< 0.01%	0.0	1.0	Yes		Di Meglio et al., 2016 [19]
52	> 16 / M	*MFN2*	c.1085C > T	p.Thr362Met	AD	No	< 0.01%	0.0	1.0	Yes		Chung et al., 2006 [42]
59	> 16 / M	*MLYCD*	c.206C > T /?	p.Ala69Val	AR	No	< 0.01%	0.07	0.685	Yes		Wightman et al., 2003 [24]
69	> 16 / F	*DGUOK*	c.750G > T /?	p.Leu250Phe	AR	No	0	0.0	1.0	No		–

Mutation Taster predicted all variants to be disease causing; *NN* neonatal, *AR* autosomal recessive, *AD* autosomal dominant, *htz* heterozygous, *NA* not available, *Csg* consanguinity

Pathogenic rare variants in *OPA3* have previously been shown to cause optic atrophy, with either autosomal dominant or autosomal recessive inheritance [22]. We identified a novel heterozygous variant in *OPA3* c.229G > A; p.Ala77Thr in a young patient (N°22) presenting with delayed motor development, refractory epilepsy with pyramidal and extrapyramidal syndrome (Table 5). This variant was inherited from his mother suggesting a recessive inheritance but we could not find the second mutation. However, the absence of optic atrophy at 9 years of age is not consistent with a causative effect of the identified variant. In the last 3 cases (N°^s^69, 59, 2), we identified possible deleterious variants in the *DGUOK*, *MLYCD* and *PC* genes, responsible for recessive diseases, in a heterozygous state (Table 5) [23–25]. Respective clinical presentations and absence of associated deletions on the controlateral alleles allowed to eliminate their involvement in the disease.

## Discussion

Since the advent of NGS, several studies have tried to evaluate the relevance of targeted gene panel sequencing and whole exome sequencing for molecular diagnosis of mitochondrial diseases. To date, responsible genes are unknown in more than 2/3 of patients affected by a mitochondrial disorder. This situation is due to the large genetic heterogeneity of these diseases. NGS has greatly improved the screening of the mitochondrial genome. Nevertheless, once the mtDNA has been eliminated, only around 250 nuclear genes out of the 1500 potentially involved are known to date. Several studies have reported a NGS approach based on targeted gene panel sequencing, on WES or both in cohorts of patients suspected of having a mitochondrial disorder [6, 26–32]. The comparison between these different studies is extremely difficult. The success of molecular diagnosis is highly dependent on the quality of the clinical diagnosis and biochemical characterization. The number of patients reported in the different cohorts ranged from 24 to 148 with heterogeneous populations in terms of ages, isolated or familial cases, biochemically proven respiratory chain deficiency or previous screening for specific sets of genes known to be associated with phenotypes. The success rate varies from 8 to 24% when gene panels are used, the higher rate belongs to a “MitoExome” (1034 nuclear DNA genes and 37 mtDNA genes) [26, 27, 29]. Results also depend on bioinformatic data processing, variant prioritization and numerous others parameters. It is from 17 to more than 50% with WES-based strategies [30, 31]. Recently, Legati and colleagues analysed 125 patients with a mitochondrial disease. Their cohort included 78 children with age of onset ≤1, the mean onset of remaining ones was 18.6 years. A previous screening had eliminated mtDNA mutations and nuclear genes known to be associated with the observed phenotypes [6]. Using a targeted gene panel including 132 genes, they identified the causative mutations in 19 patients (15.2%). They estimated a diagnostic success of the NGS panel strategy of around 25%, when used as a first strategy approach [6]. We wondered if using larger panels could improve the success rate. We studied a cohort of 80 patients highly suspected of having mitochondrial disease, including children and adults, sent to our center during the first 3 months of 2016. Abnormalities of the mitochondrial genome were found in 15 cases and we used a custom-made targeted panel including 281 genes in the 65 remaining patients. Among these genes, 266 were known to be involved in mitochondrial disorders and 15 were candidates based on their role or involvement in different pathways that include genes responsible for respiratory chain deficiency. The number of novel genes responsible for RC deficiency increases dramatically (more than 110 in the last five years) and it should be noted that the version of the panel we use today is still different from that used in this study. With the panel described we identified pathogenic variants in 8 patients out of 65 (12.3%), including 5/23 children (21.7%) and 3/42 adults (7.1%). Our cohort is a mix of pediatric and adult patients and the success rate in the pediatric population (21.7%) is higher than the one found in the cohort described by Legati and colleagues [6]. However, they had previously excluded the main candidate genes and the 2 studies are difficult to compare. We also clearly show that mtDNA abnormalities are mainly found in adult patients (25%) compared to children (4.1%) whereas the situation is reversed for pathogenic variants in nuclear genes (7.1% in adults and 21.7% in children). However, our data suggest that a gradual increase in the size of the panels could not resolve all undiagnosed cases.

WES can improve the diagnostic rate by discovering novel mitochondrial disease-linked gene. Another reason to prefer WES than panels as a first strategy approach is that patients with respiratory chain deficiency may harbor pathogenic variants in genes apparently not related to mitochondrial function and that respiratory chain abnormalities may be secondary to other disease. The patient n°64, who carries two compound heterozygous mutations in *SYNE1,* illustrates this situation (Table 4). *SYNE1* encodes a multi-isomeric modular protein which forms a network between organelles and the actin cytoskeleton to maintain the subcellular spatial organization [33]. This gene is responsible for different neuromuscular disorders including autosomal dominant Emery-Dreifuss muscular dystrophy 4 or autosomal recessive spinocerebellar ataxia 8 (SCAR 8) [34, 35]. It was not an obvious candidate gene for mitochondrial disease. However, unpublished results suggested that patients presenting with ataxia associated with *SYNE1* mutations may have a secondary mitochondrial dysfunction. It is for this reason that we included *SYNE1* in our panel leading to the identification of two variants in a patient who presented ataxia and CPEO in adulthood with mitochondrial aggregates and multiple deletions of mtDNA in muscle. Further analyses will be necessary to explain these unexpected secondary effects and to exclude an artefact. However, mutations in non-mitochondrial proteins can make finding the pathogenic variants even more difficult. Functional analyses are also required to distinguish between confirmed or possible disease-causing mutations. Familial segregation and concordant clinical phenotype are 2 critical parameters. Nevertheless, segregation studies are often impossible in adult patients and in pediatric cohorts, the family size is mostly small. Furthermore, in general mitochondrial disease displays poor correlation between genotype and phenotype.

## Conclusions

Our data highlights the great underlying genetic heterogeneity of suspected mitochondrial disease and how hard is to assign a pathogenic role to each variant identified by NGS. International networks are required to create a common genetic database in order to improve the molecular diagnosis of these diseases. Our results also confirm that a panel strategy is not optimal to identify the molecular abnormalities associated with mitochondrial disease even by increasing the number of genes analyzed. Data from the literature suggests that exome may be of greater interest than gene panels that need to be permanently reassessed based on the identification of new responsible genes. Nevertheless, further comparative studies will be needed to determine the best strategy to use for the diagnosis of these pathologies.

## Additional file

Additional file 1: **Table S1.** List of the genes included in the NGS panel. (PDF 7.44 kb)

## Abbreviations

CMT2A: Charcot-Marie-Tooth 2A
CPEO: Chronic progressive external ophtalmoplegia
EURO-NMD: European Network of reference centers for rare neuromuscular diseases
LHON: Leber hereditary optic neuropathy
LS: Leigh syndrome
MAF: Minor allele frequency
MD: Mitochondrial disorders
MtDNA: Mitochondrial DNA
nDNA: Nuclear DNA
NGS: Next generation sequencing
OXPHOS: Oxidative phosphorylation
PH: Pleckstrin homology
RC: Respiratory chain
VUS: Variant of uncertain significance
WES: Whole exome sequencing
