# Supplementary material for: Targeted next generation sequencing with an extended gene panel does not impact variant detection in mitochondrial diseases
Source: BMC Med Genet. 2018 Apr 7;19:57. doi: 10.1186/s12881-018-0568-y (PMC5889585; doi:10.1186/s12881-018-0568-y)
Supplement: Supplementary file 1 — Table S1. List of the genes included in the NGS panel. (PDF 7.44 kb) [file 12881_2018_568_MOESM1_ESM.pdf]

Oxydative phosphorylation enzymes

Complex I

NDUFA1, *NDUFS2*, *NDUFA2*, *NDUFS3*, *NDUFA9*, *NDUFS4*, *NDUFA10*, *NDUFS6*, *NDUFA11*, *NDUFS7*, *NDUFA12*, *NDUFS8*, *NDUFB3*, *NDUFV1*, *NDUFB9*, *NDUFV2*, *NDUFS1*

Complex II

SDHA, *SDHB*, *SDHC*, *SDHD*

Complex III

CYC1, *UQCRB*, *UQCRC2*, *UQCRQ*

Cytochrome c oxidase

COX4I2, *COX6B1*, *COX7B*, *TACO1*

ATP synthase

ATP5A1, *ATP5E*

Assembly

Complex I

FOXRED1, *NDUFAF1*, *NDUFAF2*, *NDUFAF3*, *NDUFAF4*, *NDUFAF5*, *NDUFAF6*

Complex II

SDHAF1, *SDHAF2*

Complex III

BCS1L, *TTC19*

Cytochrome c oxidase

CEP89, *COA5*, *COX10*, *COX14*, *COX15*, *COX20*, *PET100*, *SURF1*

ATP synthase

ATPAF2, *TMEM70*

Cofactors

FeS clusters

BOLA3, *NUBPL*, *FDX1L*, *FXN*, *GLRX5*, *IBA57*, *ISCU*, *LYRM4*, *NFU1*

Cu

SCO1, *SCO2*

Coenzyme A

COASY, *PANK2*

Biotin

BTD, *HLCS*

Heme

ALAS2, *PPOX*, *CYCS*, *HCCS*

Thiamine

TPK1

Lipoic Acid

LIAS, *DLD*

Fe

SFXN4

Enzymes

Fatty acid oxydation

ACAD8, *CPT2*, *ACAD9*, *HADH*, *ACADM*, *HADHA*, *ACADS*, *HADHB*, *ACADSB*, *ETFA*, *ACADVL*, *ETFB*, *CPT1A*, *ETFDH*

Krebs cycle

ACO2, *PC*, *FH*, *PCCA*, *IDH3B*, *PCCB*, *MDH2*, *PCK2*

Ketone bodies

ACAT1, *HMGCL*, *HMGCS2*

Coenzyme Q

COQ2, *COQ9*, *COQ6*, *PDSS1*, *COQ8A*, *PDSS2*, *COQ8B*

Pyruvate dehydrogenase

DLAT, *PDHX*, *PDHA1*, *PDK3*, *PDHB*, *PDP1*

Aldehyde dehydrogenase

ALDH4A1, *ALDH6A1*

Others

AK2, *GK*, *AMACR*, *GLDC*, *AMT*, *GLUD1*, *ATL1*, *HIBCH*, *AUH*, *HK1*, *BCAT2*, *HOGA1*, *BCKDHA*, *IDH1*, *BCKDHB*, *IDH2*, *CPS1*, *IVD*, *CRAT*, *L2HGDH*, *CYB5R3*, *MAOA*, *CYP27A1*, *MCCC1*, *DDHD1*, *MCCC2*, *D2HGDH*, *MCEE*, *DBT*, *MLYCD*, *DECR1*, *MMAA*, *DHODH*, *MMAB*, *DMGDH*, *MMADHC*, *ETHE1*, *MUT*, *FASTKD2*, NAGS, *FBP1*, *OAT*, *GAMT*, *OGDH*, *GATM*, *OTC*, *GCDH*, *OXCT1*, *GCK*, *PNPLA2*, *GCSH*, *WWOX*

DNA, RNA, and protein synthesis

Replication

DNA2, *MGME1*, *POLG*, *POLG2*, *RMRP*, *TWNK*

Nucleotides

DGUOK, *MPV17*, *RRM2B*, *SLC25A4*, *SUCLA2*, *SUCLG1*, *TK2*, *TYMP*

RNA metabolism

ELAC2, *HSD17B10*, *MTPAP*, *LRPPRC*, *MTO1*, *PNPT1*, *PUS1*, *TRMU*

Translation regulation

GFM1, *C12orf65*, *RMND1*, *TSFM*, *TUFM*

Ribosomes

MRPL3, *MRPL44*, *MRPS16*, *MRPS22*

tRNA synthetases

AARS2, *DARS2*, *EARS2*, *FARS2*, *GARS*, *HARS2*, *KARS*, *LARS2*, *MARS2*, *MTFMT*, *RARS2*, *SARS2*, *YARS2*

Repair

ERCC6, *APTX*, *UNG*

Homeostasis

Protein Import

AIFM1, *GFER*, *XPNPEP3*

Transporter

ABCB7, *SLC25A15*, *DNAJC19*, *SLC25A19*, *MPC1*, *SLC25A20*, *SLC19A2*, *SLC25A22*, *SLC19A3*, *SLC25A3*, *SLC22A5*, *SLC25A38*, *SLC25A1*, *SLC33A1*, *SLC25A12*, *SLC6A8*, *SLC25A13*, *TIMM8A*

Protein quality

AFG3L2, *PARK2*, *CLPP*, *SACS*, *HSPD1*, *SPG7*, *HTRA2*

Fission and fusion

DNM1L, *MFN2*, *GDAP1*, *OPA1*

Lipid

AGK, *SERAC1*, *TAZ*

MICOS

CHCHD10

Ca2+

CISD2, *WFS1*

Trafficking

KIF5A, *KIF1B*, *SPG20*

Apoptosis

DIABLO

Unclear function

OPA3, *SAMHD1*, *PNKD*, *SPAST*, *PARK7*, *STAR*, *PINK1*, *TMEM126A*, *REEP1*, *YWHAE*

Candidate genes

CHCHD3, *KIF13A*, *CHCHD4*, *MFN1*, *CHCHD6*, *MINOS1*, *ECHS1*, *PACS2*, *FBXL4*, *PIF1*, *FIS1*, *SUCLG2*, *GTPBP3*, *SYNE1*, *IMMT*
